# Functional fiber supplementation improves reproductive performance in sows by reshaping gut microbial functions related to immunity and metabolic health

**DOI:** 10.3389/fvets.2026.1834008

**Published:** 2026-05-07

**Authors:** Shengnan Yin, Shiting Fu, Jinghua Cheng, Mu Wang, Yuanfei Zhou, Hongkui Wei, Jian Peng

**Affiliations:** 1 College of Animal Science and Technology, Huazhong Agricultural University, Wuhan, China; 2 Frontiers Science Center for Animal Breeding and Sustainable Production, Wuhan, China; 3 The Cooperative Innovation Center for Sustainable Pig Production, Wuhan, China

**Keywords:** dietary fiber, microbial functions, microbiota, reproductive performance, sow

## Abstract

**Introduction:**

Dietary fiber has been reported to regulate reproductive performance, yet its role in microbial functions during late gestation remains unclear. This study aimed to explore the effect of functional fiber supplementation on the gut microbiome and reproductive performance of sows in late gestation, as well as the potential underlying mechanisms.

**Methods:**

A subset of sows from a large-scale feeding trial was selected and assigned to either a control diet (CON, *n* = 501) or a functional fiber-supplemented diet (DF, *n* = 409). Relevant indicators including reproductive performance, plasma physiological parameters, and gut microbiome were detected and analyzed.

**Results:**

Compared with the CON group, DF treatment significantly increased the numbers of total born, born alive and healthy piglets (*P* < 0.05). Sows in the DF group exhibited higher plasma IL-10 levels, lower plasma reactive oxygen species, reduced insulin resistance and improved insulin sensitivity (higher HOMA-IS) (*P* < 0.05). Microbiome analyses revealed enrichment of *NK4A214_group* associated taxa and suppression of Treponema-dominated communities. Further functional profiling showed increased antigen processing and presentation, estrogen signaling pathway, IL-17 signaling pathway, progesterone-mediated oocyte maturation and Th17 cell differentiation pathways, alongside decreased biofilm formation – Pseudomonas aeruginosa pathways. These microbial changes were associated with improved reproductive performance.

**Discussion:**

Taken together, these results indicate that functional fiber improves reproductive outcomes by functionally remodeling the gut microbiome toward enhanced immune regulation and metabolic homeostasis during late gestation, providing a theoretical basis for the application of functional fiber in late gestation sow feeding.

## 1 Introduction

Gestation and lactation are critical physiological periods in sows, characterized by profound metabolic and immunological adaptations (1). During late gestation, the rapid growth of fetuses markedly increases maternal energy and nutrient demands, placing sows under high metabolic pressure (1). At this stage, sows are prone to reduced insulin sensitivity, disturbed glucose and lipid metabolism, elevated oxidative stress, persistent low-grade inflammation, and impaired intestinal barrier integrity (2–4). These physiological disturbances can contribute to constipation, intrauterine growth retardation, abortion, increased numbers of weak-born piglets, and reduced litter size, thereby compromising reproductive efficiency and causing substantial economic losses in pig production (5–7).

Accumulating evidence indicates that these physiological disturbances are closely linked to alterations in the gut microbiota, which plays a central role in regulating nutrient metabolism, immune homeostasis, intestinal barrier integrity, and susceptibility to metabolic disorders (3, 8–10). Increasingly, the gut microbiota is being recognized as a key contributor to sow reproductive physiology, with potential links to fertility, pregnancy maintenance, farrowing performance, and lactation efficiency (3, 11, 12). Gut microbial dysbiosis during gestation has been associated with unfavorable reproductive outcomes, whereas a more balanced microbial ecosystem appears to support maternal health and reproductive success (13). However, most previous studies in sows have primarily described taxonomic changes in the gut microbiota, while the functional shifts of the microbiome and their relevance to reproductive outcomes remain insufficiently understood.

Dietary fiber, defined as carbohydrates resistant to host digestion, has received increasing attention as a nutritional strategy to improve intestinal health, metabolic regulation, and immune function (14–18). Recent studies have demonstrated that dietary fiber reshapes the gut microbiota, and these fiber-induced microbial alterations are significantly associated with improved reproductive performance and metabolic homeostasis in sows (19, 20). For example, high-fiber diets during gestation have been reported to increase beneficial bacteria and reduce potentially harmful taxa, accompanied by alleviated systemic inflammation and improved defecation or farrowing-related outcomes (18, 21). These findings suggest that dietary fiber may influence sow reproductive performance, at least in part, through microbiota-mediated mechanisms. Nevertheless, important knowledge gaps remain. In particular, it is still unclear how dietary fiber alters the functional potential of the gut microbiome, which microbial functions are associated with maternal metabolic and immune regulation, and how these functional shifts are linked to reproductive performance during late gestation.

Therefore, the present study used metagenomic analysis to investigate the effects of dietary fiber supplementation during gestation on reproductive performance, metabolic and immune status, and gut microbiome characteristics in sows, with particular emphasis on microbial functional alterations during late gestation. By integrating microbial functional profiles with host physiological and reproductive indicators, this study sought to clarify the microbiome-mediated mechanisms linking dietary fiber supplementation to reproductive outcomes.

## 2 Materials and methods

### 2.1 Animal treatment and experimental diets

The animal study was reviewed and approved by the Animal Care and Use Committee of Huazhong Agricultural University (202504060003). The samples analyzed in this study were obtained from a previously established sow feeding trial conducted at a commercial pig farm in Xinyang of Tecon Biology Co, China, as previously described (22). Briefly, a total of 1,000 healthy sows (DanBred Landrace × DanBred Yorkshire, parities 1–2) were randomly assigned after artificial insemination to two dietary treatments: a control diet (CON, *n* = 501), with beet pulp and barley as the primary fiber sources, and a diet supplemented with 1% functional fiber (DF, *n* = 499). The functional fiber was a defined blend consisting of 85.7% resistant starch (Hangzhou, China) and 14.3% guar gum (Yunzhou, China), and provided by Wuhan Fanbo Biotechnology Co., Ltd. (Wuhan, China). The DF diet was formulated by partially replacing energy-yielding ingredients in the control diet while maintaining comparable net energy levels between treatments. All experimental diets were formulated to meet or exceed the nutrient requirements for gestating sows according to the NRC (2012; Supplementary Table S1). Sows were housed individually in gestation stalls (2.2 m × 0.65 m × 0.6 m) under standard commercial management conditions. During gestation, sows were fed restricted amounts according to gestational stage: 2.6 kg/d from days 0 to 30, 2.2 kg/d from days 31 to 90, and 2.8 kg/d from days 91 to 109. From day 110 until the day before farrowing, feed allowance was adjusted to 2.0 kg/d. The farrowing room temperature was maintained at approximately 20–22 C, and water was freely available to sows and piglets throughout the experimental period. All animals were managed under the same routine husbandry conditions by the same farm personnel to reduce environmental and management-related variation. All sows received the farm's routine vaccinations, including anti-diarrheal and seasonal immunizations. Any sow treated with antibiotics during the reproductive cycle corresponding to the sampling period was excluded from sample collection.

### 2.2 Measurements of reproductive performance

Sow performance and reproductive parameters were recorded during gestation and lactation. These included total piglets born, live born piglets, birth weight, stillbirths, and mummified fetuses. Reproductive performance data were collected, encompassing the number of total piglets born, piglets born alive, healthy piglets (birth weight ≥ 0.8 kg), piglets with intrauterine growth retardation (IUGR; birth weight <0.8 kg), piglets born dead (stillborn, mummified, crushed, or abnormal), litter birth weight, and individual piglet birth weight. White stillbirths were defined as piglets that died during parturition or shortly before farrowing, characterized by a pale or normal skin color and the absence of obvious autolysis. Black stillbirths were defined as piglets that died before the onset of farrowing and showed dark discoloration of the skin, indicating decomposition/autolysis due to a longer intrauterine retention time. Mummified fetuses were identified as fetuses that died earlier during gestation and were dehydrated, shrunken, and dark brown to black in appearance.

### 2.3 Sample collection

The present study was derived from the same animal experiment described in our previous report (22). Data on sow reproductive performance were recorded during gestation and at farrowing.

In the original study, 20 sows per group were sampled, and 16 sows per group were selected for 16S rRNA gene sequencing. In the current study, to further investigate the effects of dietary intervention at a key prepartum stage, fecal and plasma samples collected on gestation day 109 were selected for downstream analyses. Among the 16 sows per group previously analyzed by 16S rRNA gene sequencing, 10 sows per group with complete sample availability and representative production performance were selected for integrated 16S rRNA gene sequencing, shotgun metagenomic sequencing, and plasma biochemical analyses.

Prior to parturition, fecal samples were collected by rectal grab sampling. The central portion of each fecal sample was transferred into a sterile 25 mL tube and immediately snap-frozen in liquid nitrogen, followed by storage at −80 C until analysis. Blood samples were collected by ear venipuncture into 10 mL anticoagulant tubes. Plasma was separated by centrifugation at 3,500 rpm for 10 min at room temperature and then stored at −80 C until analysis.

### 2.4 Chemical analyses

Plasma and fecal concentrations of inflammatory cytokines, including interleukin-6 (IL-6) and interleukin-10 (IL-10), as well as lipocalin-2 (LCN2) and lipopolysaccharide (LPS) were quantified using commercially available enzyme-linked immunosorbent assay kits (Bio-Camio Co. Ltd., Nanjing, China). Oxidative status was evaluated by determining reactive oxygen species (ROS), malondialdehyde (MDA), and total superoxide dismutase (T-SOD) levels with corresponding biochemical assay kits (Nanjing Jiancheng Bioengineering Institute, Nanjing, China). Plasma glucose was determined with a glucose dehydrogenase activity colorimetric assay kit (BioVision Inc., CA, United States). All procedures were performed following the manufacturers' recommended protocols.

### 2.5 Short-Chain fatty acids determination

Fecal SCFA concentrations were measured by gas chromatography according to Yang et al. (23) with slight modifications. In brief, 0.5 g of feces was homogenized in 1 mL of deionized water and centrifuged at 12,000 × g for 10 min at 4 C. An aliquot of the supernatant (0.1 mL) was acidified with 25% metaphosphoric acid (5:1, v/v) on ice for 30 min, followed by centrifugation at 12,000 × g for 10 min. The resulting supernatant was mixed with an equal volume of ethyl acetate and centrifuged again at 12,000 × g for 10 min at 4 C. The upper phase was collected for chromatographic analysis.

Chromatographic analysis was performed on a GC 3,000 series gas chromatograph (Thermo, Waltham, MA, USA) equipped with an Omegawax 250 column (30.0 m × 0.25 mm × 0.25 μm; Sigma, St. Louis, MO, USA) and an iConnect™ flame ionization detector (FID). Samples were introduced in split mode at an injector temperature of 240 C, with a column flow of 1.0 mL/min and a split flow of 5.0 mL/min. The oven temperature was programmed as follows: 75 C for 1 min, ramped to 140 C at 20 C/min, then to 180 C at 8 C/min, and finally to 240 C at 15 C/min with a final hold of 5 min.

SCFAs were identified by comparison with the retention times of authentic standards and quantified using standard calibration curves. The concentrations of acetate, propionate, butyrate, isobutyrate, valerate, and isovalerate were reported as μmol/g feces.

### 2.6 16S rRNA gene sequencing and bioinformatics analysis

DNA extraction, 16S rRNA gene sequencing, and quality control were performed as described in our previous study (22). Briefly, microbial genomic DNA was extracted from fecal samples using a commercial stool DNA extraction kit (Qiagen, Germany) according to the manufacturer's instructions. The kit included PowerBead tubes for cell disruption. The V3–V4 region of the bacterial 16S rRNA gene was amplified using primers 338F and 806R for 27 PCR cycles. The PCR amplicons were purified by agarose gel extraction using the AxyPrep DNA Gel Extraction Kit (Axygen Biosciences, Union City, CA, USA) according to manufacturer's instructions, followed by paired-end sequencing on an Illumina MiSeq PE300 platform (Illumina, San Diego). Sequence processing and amplicon sequence variant (ASV) inference were conducted using the QIIME2 pipeline (24) with the DADA2 algorithm (25). Taxonomic classification was performed with a Naive Bayes classifier trained on the SILVA 16S rRNA database (v138).

### 2.7 Metagenomic sequencing and bioinformatic analysis

Fecal DNA samples collected on gestation day 109 (*n* = 20) were subjected to shotgun metagenomic sequencing. Prior to library construction, DNA quality was assessed, and only samples with an OD260/280 ratio of 1.8–2.0 were used for subsequent analysis. DNA was fragmented to an average size of ~400 bp using Covaris M220 (Gene Company Limited, China), and paired-end libraries were constructed using NEXTflex™ Rapid DNA-Seq kit (Bioo Scientific, Austin, TX, USA). Paired-end sequencing (150 bp) was performed on the DNBSEQ-T7 platform (Wefind Biotechnology Co., Ltd., Wuhan, China) according to the manufacturer's instructions.

Raw sequencing reads were quality-filtered using fastp (v0.20.0) (26) to remove adapters, low-quality bases (minimum Q20), and reads shorter than 50 bp.

Host-derived reads were removed by aligning to the swine genome (NCBI assembly accession: GCF_000003025.6) using BWA (27), and the remaining reads were retained as clean reads. In total, 503.67 Gb of raw data were generated from the 20 samples. After quality filtering and host-read removal, 482.06 Gb of clean data were obtained, with an average of 24.10 Gb per sample (range, 17.68–28.34 Gb). The Q20 values of the clean reads ranged from 98.41 to 99.00%, with an average of 98.79%, while the Q30 values ranged from 93.93 to 96.56%, with an average of 95.49%.

High-quality reads were *de novo* assembled into contigs using MEGAHIT (v1.2.9) (28) with k-mer sizes of 45, 71, 99, and 121, and contigs ≥500 bp were retained for downstream analysis. Open reading frames (ORFs) were predicted from the assembled contigs using Prokka (v1.14) (29). The predicted protein sequences were then annotated against the Kyoto Encyclopedia of Genes and Genomes (KEGG) database using DIAMOND in BLASTP mode with an e-value threshold of 1e-5. KEGG pathway abundance was subsequently summarized for downstream differential pathway analysis.

For metagenome-assembled genome (MAG) reconstruction, contigs were binned using MetaBAT2 (30) based on sequence composition and coverage across samples. MAG quality was evaluated using CheckM, and high-quality MAGs (completeness ≥ 70% and contamination ≤ 10%) were retained, yielding 2,702 MAGs. These high-quality MAGs were further dereplicated using dRep at a 99% average nucleotide identity (ANI) threshold to generate a non-redundant set of 1,721 MAGs for downstream analyses. MAGs were taxonomically classified using the GTDB-Tk v0.3.2 (Genome Taxonomy Database Toolkit) (31).

### 2.8 Statistical analysis

GraphPad Prism (version 7, GraphPad Software, USA) was used for the graphical representation of the reproductive performance and measured biochemical parameters. Data are presented as mean ± standard deviation (SD). Microbial data from 16S rRNA gene sequencing and metagenomic sequencing were analyzed in the R statistical interface (www.r-project.org). Before analysis, Shapiro-Wilk and Levene tests were performed for the normality and heteroscedasticity of continuous data (with the significance level set at 5%). When the assumptions for parametric tests were not satisfied, non-parametric tests were applied.

The MicrobiotaProcess package in R (Version 1.6.6) (32) was employed for alpha diversity analyses, the Vegan package (version 2.6-8) and ggplot2 (version 3.5.1) were performed to calculate beta diversity and visualized plot. In addition, differentially abundant genera between groups were identified using linear discriminant analysis effect size (LEfSe) (33). LEfSe analysis was performed on the Wekemo Bioincloud platform (https://www.bioincloud.tech) following the standard workflow described previously (34), with default parameters unless otherwise specified. In addition, microbial differential abundance analysis between groups was performed using the Wilcoxon rank-sum test.

Correlation analysis was conducted using Spearman's rank correlation. This non-parametric approach was selected because it is suitable for analyzing variables that do not follow a normal distribution (the core prerequisite for parametric Pearson's correlation), is highly robust to outliers and extreme values frequently observed in microbial datasets, and can reliably detect monotonic associations between variables regardless of whether the relationship is strictly linear. *P* values were adjusted for multiple comparisons using the false discovery rate (FDR) method. Potential confounding factors, such as parity and initial birth weight, were not included as covariates in the present correlation analysis.

For functional analysis, differential KEGG pathway was assessed using generalized linear models (GLM) implemented in the broom package (v1.0.11) in *R*. The *p* values obtained from the models were adjusted for multiple testing using the Benjamini–Hochberg method to control the false discovery rate (FDR). Statistical significance was determined at *P* < 0.05, or adjusted *P* < 0.05.

For KEGG functional analysis, abundance values for each KEGG level C pathway were transformed as log10 (abundance + 1) prior to analysis. Differential pathway abundance between groups was assessed using generalized linear models (GLM) fitted in *R*, with log-transformed pathway abundance as the response variable and group as the explanatory variable. A Gaussian distribution with an identity link was used, and the model formula was log_abundance ~ group. The coefficient and *p* value for the group effect were extracted using the broom package (v1.0.11). *P* values were adjusted for multiple testing using the Benjamini–Hochberg method to control the false discovery rate (FDR). Statistical significance was determined at *P* < 0.05 or adjusted *P* < 0.05.

## 3 Results

### 3.1 Performance in sows

As shown in Table 1, the number of total born was significantly improved (*P* < 0.0001) in the DF group compared to the CON group. The numbers of born alive and healthy piglets were also significantly improved (*P* < 0.01) in the DF group. Furthermore, dietary supplementation with DF led to a significant increase in litter birth weight (*P* < 0.05). Additionally, there were no significant differences in the number of IUGR, white stillbirths, black stillbirths, mummified fetuses, abnormal piglets, and individual average piglet weight (*P* > 0.05).

**Table 1:** Effects of dietary supplementation with DF on litter performance of sows.

Item	CON	DF	*P*-value
No. of sows	10	10	
Parity	2	2	
Total born, *n*	15.502 ± 0.73	20.901 ± 0.64	*P <* 0.01
Born alive, *n*	14.602 ± 0.37	18.102 ± 0.17	*P <* 0.01
Healthy piglets, *n*	14.002 ± 0.24	17.702 ± 0.10	*P <* 0.01
IUGR, *n*	0.600 ± 0.91	0.400 ± 0.66	0.72
White stillbirth, *n*	0.700 ± 0.90	1.701 ± 0.42	0.11
Black stillbirth, *n*	0.100 ± 0.30	0.801 ± 0.25	0.21
Number of mummified fetus	0	0.200 ± 0.60	0.99
Number of abnormal piglet	0.100 ± 0.30	0.100 ± 0.30	0.99
Litter birth weight, kg	18.442 ± 0.18	21.773 ± 0.52	*P <* 0.05
Individual average weight, kg	1.280 ± 0.14	1.200 ± 0.11	0.22

CON, control diet; DF, functional fiber diet. IUGR, intrauterine growth retardation.

Data are expressed as means ± SD. Differences were considered significant at *P* < 0.05.

### 3.2 Inflammatory response in sows

We examined the levels of inflammation and anti-inflammatory biomarkers, including LPS, IL-6, IL-10, and LCN2, in fecal and plasma samples (Tables 2, 3). No significant differences in LPS and IL-6 concentrations were observed in either plasma or fecal samples between the two groups (*P* > 0.05). Additionally, fecal LCN2 levels showed no significant difference between treatments (*P* > 0.05). Although IL-10 levels in feces were numerically higher in the DF group than in the CON group, no significant difference was observed (*P* > 0.05). However, IL-10 levels in plasma were significantly higher in the DF group than in the CON group (*P* < 0.05).

**Table 2:** Effects of dietary supplementation with DF on fecal inflammatory markers of sows.

Item	CON	DF	*P*-value
LPS, ng/g	166.563 ± 6.21	152.862 ± 4.30	0.36
IL-6, pg/g	1,367.669 ± 4.00	1,230.783 ± 72.85	0.30
IL-10, pg/g	708.787 ± 7.98	744.929 ± 4.34	0.43
LCN2, ng/g	49.691 ± 1.52	52.461 ± 9.35	0.20

CON, control diet; DF, functional fiber diet. LPS, lipopolysaccharide; IL-6, interleukin-6; IL-10, interleukin-10; LCN2, lipocalin-2.

Data are expressed as means ± SD. n = 10. Differences were considered significant at *P* < 0.05.

**Table 3:** Effects of dietary supplementation with DF on plsma inflammatory markers of sows.

Item	CON	DF	*P*-value
LPS, ng/ml	305.588 ± 1.40	272.667 ± 1.17	0.37
IL-6, pg/ml	233.063 ± 5.03	224.362 ± 2.44	0.56
IL-10, pg/ml	117.402 ± 1.85	158.514 ± 1.81	*P* < 0.05

CON, control diet; DF, functional fiber diet. LPS, lipopolysaccharide; IL-6, interleukin-6; IL-10, interleukin-10; LCN2, lipocalin-2.

Data are expressed as means ± SD. n = 10. Differences were considered significant at *P* < 0.05.

### 3.3 Oxidative status and insulin homeostasis in sows

Tables 4, 5 present the results relating to variables associated with oxidative stress and glucose metabolism. As shown in Table 4, no significant differences were found in the plasma levels of MDA and T-SOD between the two groups (*P* > 0.05). However, compared to the CON group, plasma ROS levels were significantly lower in the DF group (*P* < 0.05). Regarding glucose metabolism (Table 5), dietary supplementation with DF did not significantly affect fasting glucose and insulin levels (*P* > 0.05). Nevertheless, the HOMA-IR index was significantly decreased (*P* < 0.05), and the HOMA-IS index was significantly increased (*P* < 0.05) in the DF group compared to the CON group.

**Table 4:** Effects of dietary supplementation with DF on oxidative stress markers of sows.

Item	CON	DF	*P*-value
MDA, mmol/ml	8.775 ± 0.12	6.421 ± 0.45	0.26
T-SOD, U/ml	191.184 ± 4.13	180.302 ± 4.63	0.58
ROS, U/ml	7.091 ± 0.93	4.551 ± 0.85	*P* < 0.05

CON, control diet; DF, functional fiber diet; MDA, malondialdehyde; T-SOD, total superoxide dismutase; ROS, reactive oxygen species.

Data are expressed as means ± SD. n = 10. Differences were considered significant at *P* < 0.05.

**Table 5:** Effects of dietary supplementation with DF on glucose metabolism of sows.

Item	CON	DF	*P*-value
Fasting glucose	4.670 ± 0.48	4.440 ± 0.48	0.37
Insulin	12.642 ± 0.89	11.932 ± 0.38	0.58
HOMA-IR	2.290 ± 0.22	1.840 ± 0.39	*P* < 0.05
HOMA-IS	0.0200 ± 0.002	0.0240 ± 0.004	*P* < 0.05

CON, control diet; DF, functional fiber diet; HOMA-IR, homeostasis model assessment of insulin resistance; HOMA-IS, homeostasis model assessment of insulin sensitivity.

Data are expressed as means ± SD. n = 10. Differences were considered significant at *P* < 0.05.

### 3.4 Fecal short-chain fatty acids in sows

**Figure 1:**
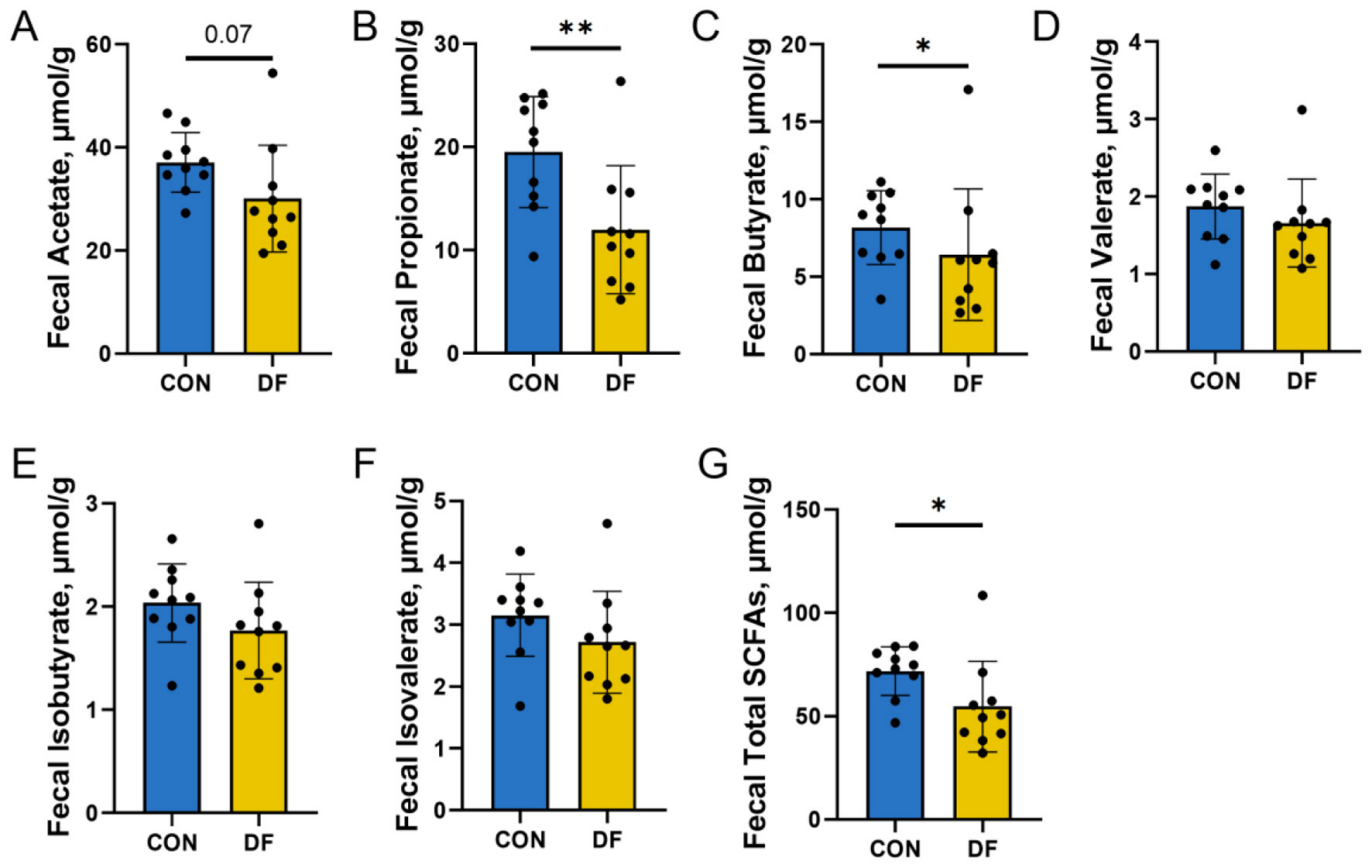
Effects of DF on fecal SCFA concentrations of sows: **(A)** Acetate; **(B)** Propionate; **(C)** Butyrate; **(D)** Valerate; **(E)** Isobutyrate; **(F)** Isovalerate; **(G)** Total SCFAs. CON, control diet; DF, functional fiber diet. Data are presented as mean ± SD. *n* = 10. *P* < 0.05 indicates statistical significance (*P < 0.05, **P < 0.01).

The concentrations of fecal short-chain fatty acids (SCFAs) were measured to assess the impact of functional fiber supplementation. Compared with the CON group, the DF group exhibited a significantly lower concentration of total fecal SCFAs (*P* < 0.05; Figure 1G). Specifically, the concentrations of propionate (*P* < 0.01; Figure 1B) and butyrate (*P* < 0.05; Figure 1C) were significantly reduced in the DF group. Additionally, a downward trend was observed in the fecal acetate concentration of the DF group (*P* = 0.07; Figure 1A). No significant differences were observed between the two groups in the concentrations of valerate, isobutyrate, and isovalerate (Figures 1D–F).

### 3.5 Microbial diversity and composition in sows

**Figure 2:**
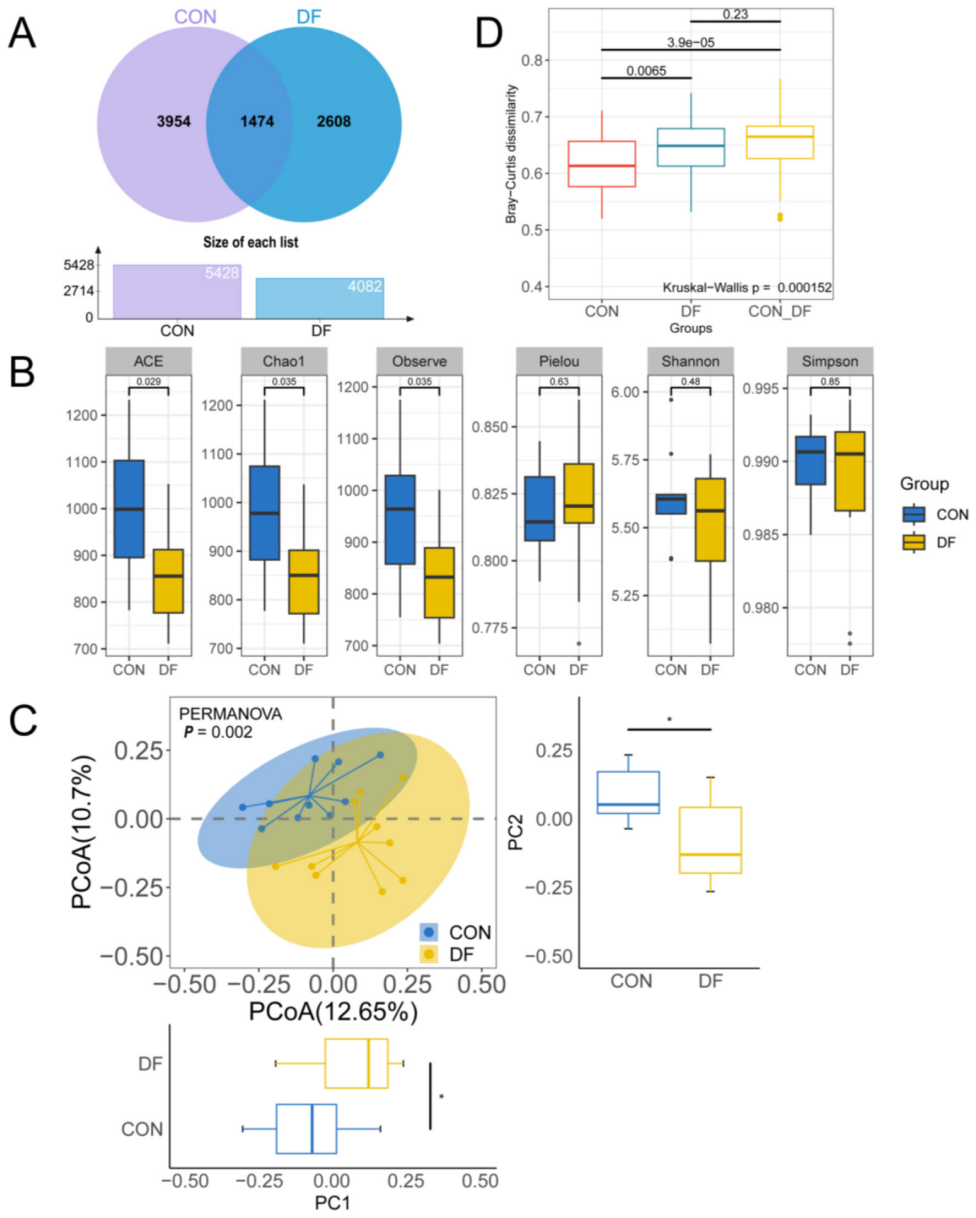
Effects of DF on fecal microbial diversity of sows: **(A)** the amplicon sequence variants (ASVs) in sow feces, **(B)** alpha diversity, **(C, D)** beta diversity based on Bray-Curtis distances. CON, control diet; DF, functional fiber diet. Results are expressed as median and quartile. *P* < 0.05 indicates statistical significance (*P < 0.05).

16S rRNA gene sequencing was conducted to study the microbial diversity and composition in the feces of sows in the two groups. As shown in Figure 2A, there were 5,428 and 4,082 ASVs obtained from the CON and DF groups, and 1,473 ASVs were observed in both two groups. Alpha diversity analyses revealed that the ACE, Chao1 and Observed index were significantly lower in the DF group (Figure 2B). Regarding beta diversity, principal coordinate analysis (PCoA) based on Bray-Curtis dissimilarity showed distinct separation of the microbial communities in the two groups (PERMANOVA: *P* = 0.002, Figure 2C). PC1 and PC2 analyses revealed significant differences between the two groups (*P* < 0.05, Figure 2C). As shown in Figure 2D, Kruskal–Wallis analysis revealed a significant difference in intergroup variation among the CON, DF and CON to DF (*P* = 0.000152).

**Figure 3:**
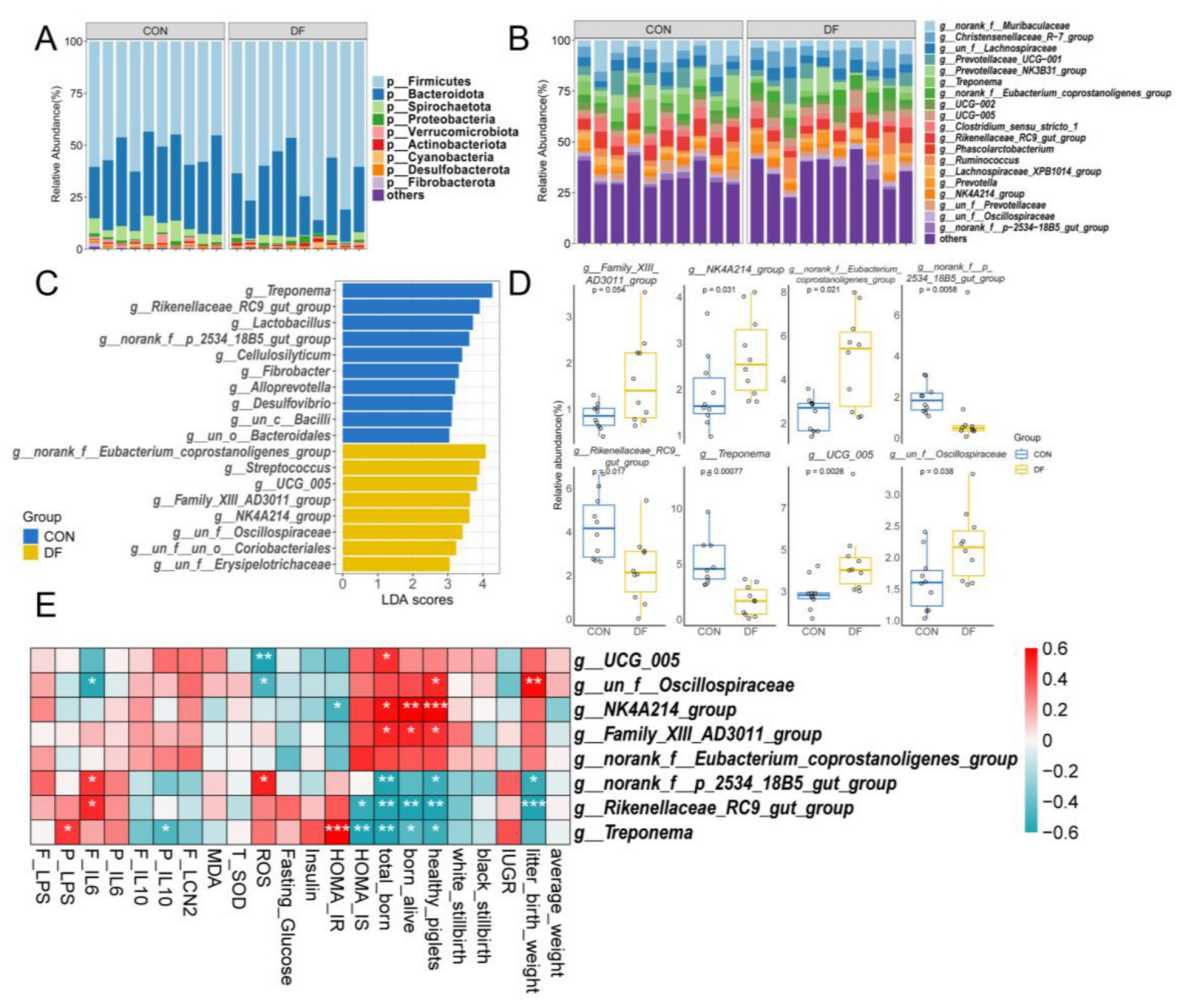
Effects of DF on fecal microbial composition of sows: **(A)** Phylum level, **(B)** Genus level, **(C)** LDA distribution and LEfSe analysis identifying genera with significant intergroup differences (LDA scores > 3), **(D)** Boxplot of 8 differential genera with relative abundance > 1%, **(E)** Relationships between the differential taxa identified in panel D and fecal and plasma parameters, as well as reproductive performance of sows. CON, control diet; DF, functional fiber diet. Color intensity represents the correlation coefficient (ρ). Results are expressed as median and quartile. *P* < 0.05 indicates statistical significance (*P < 0.05, **P < 0.01, and ***P < 0.001).

In terms of microbial composition, the fecal microbiota of sows was found to be predominantly composed of the Firmicutes and Bacteroidota (Figure 3A), and the relative abundances of the top 5 phyla were Firmicutes (59.23%) and Bacteroidota (32.02%), followed by Spirochaetota (4.02%), Proteobacteria (1.17%) and Verrucomicrobiota (0.81%). At the genus level, the most abundant taxa included *norank_f__Muribaculaceae* (6.07%), *Christensenellaceae_R-7_group* (5.66%), *un_f__Lachnospiraceae* (4.89%), *Prevotellaceae_UCG-001* (4.27%) and *Prevotellaceae_NK3B31_group* (4.23%; Figure 3B). Interestingly, the Firmicutes was significantly less abundant in the DF group than in the CON group, but there was no significant difference in the abundance of the Bacteroidota (Supplementary Figure S1). Additionally, the abundance of Spirochaetota was significantly higher in the CON group (Supplementary Figure S1).

Linear discriminant analysis (LDA) coupled with effect size estimation (LEfSe) analysis was then used to identify the bacterial taxa that were differentially abundant in the DF and CON groups (Figure 3C and E, Supplementary Figure S2A, B). The CON group showed an enrichment of the following genera: *Treponema, Rikenellaceae_RC9_gut_group, Lactobacillus, norank_f__p_2534_18B5_gut_group, Cellulosilyticum, Fibrobacter, Alloprevotella*, and *Desulfovibrio* (Figure 3C and Supplementary Figure S2A). Conversely, the DF group was enriched in *norank_f__Eubacterium_coprostanoligenes_group, Streptococcus, UCG_005, Family_XIII_AD3011_group* and *NK4A214_group* (Figure 3C). The genera with relative abundance > 1% were shown in Figure 3D. The results of LDA scores > 3 at the family level were shown in Supplementary Figure S2A. The families with a relative abundance of over 1% are shown in Supplementary Figure S2B. In summary, Oscillospiraceae, Ruminococcaceae, Anaerovoracaceae and Eubacterium_coprostanoligenes_group were significantly higher in the DF group than in the CON group (Supplementary Figure S2B). In Contrast, the p_2534_18B5_gut_group and the Spirochaetaceae were significantly higher in the CON group (Supplementary Figure S2B).

As demonstrated in our previous work (22), the late gestation period is a critical window characterized by profound gut microbiota remodeling, which is essential for maternal metabolic adaptation and optimal fetal growth (9, 11). A Spearman correlation analysis was conducted to explore the associations between the high-abundance differential genera and sow reproductive performance, as well as fecal and plasma parameters (Figure 3E). Notably, the *Treponema, norank_f__p_2534_18B5_gut_group* and *Rikenellaceae_RC9_gut_group* were negatively correlated with the numbers of total born and healthy piglets, *norank_f__p_2534_18B5_gut_group* and *Rikenellaceae_RC9_gut_group* were also negatively correlated with litter birth weight. In contrast, the *NK4A214_group* and *Family_XIII_AD3011_group* showed positive correlations with the numbers of total born, born alive and healthy piglets. Regarding metabolic and inflammatory indicators, the *Treponema* was positively correlated with HOMA-IR and plasma LPS, while being negatively correlated with HOMA-IS and plasma IL-10. Similarly, the *Rikenellaceae_RC9_gut_group* exhibited a positive association with fecal IL-6 and a negative association negatively correlated with HOMA-IS. Furthermore, *norank_f__p_2534_18B5_gut_group* was positively correlated with fecal IL-6 and ROS. Conversely, *NK4A214_group* was negatively correlated with HOMA-IR, and both *UCG-005* and *un_f__Oscillospiraceae* showed strong negative correlations with ROS levels. To further validate the robustness of the genus-level associations, correlation analysis was also performed at the family level, with the results presented in Supplementary Figure S2C. Overall, the family-level correlation patterns were largely consistent with those observed at the genus level. Taxa corresponding to Spirochaetaceae and p_2534_18B5_gut_group displayed correlation trends consistent with their representative genera, showing negative associations with litter size and insulin sensitivity–related parameters (Supplementary Figure S2C). In contrast, families including beneficial taxa such as Oscillospiraceae and Anaerovoracaceae exhibited positive correlations with reproductive outcomes (Supplementary Figure S2C).

### 3.6 Microbial functions in sows

**Figure 4:**
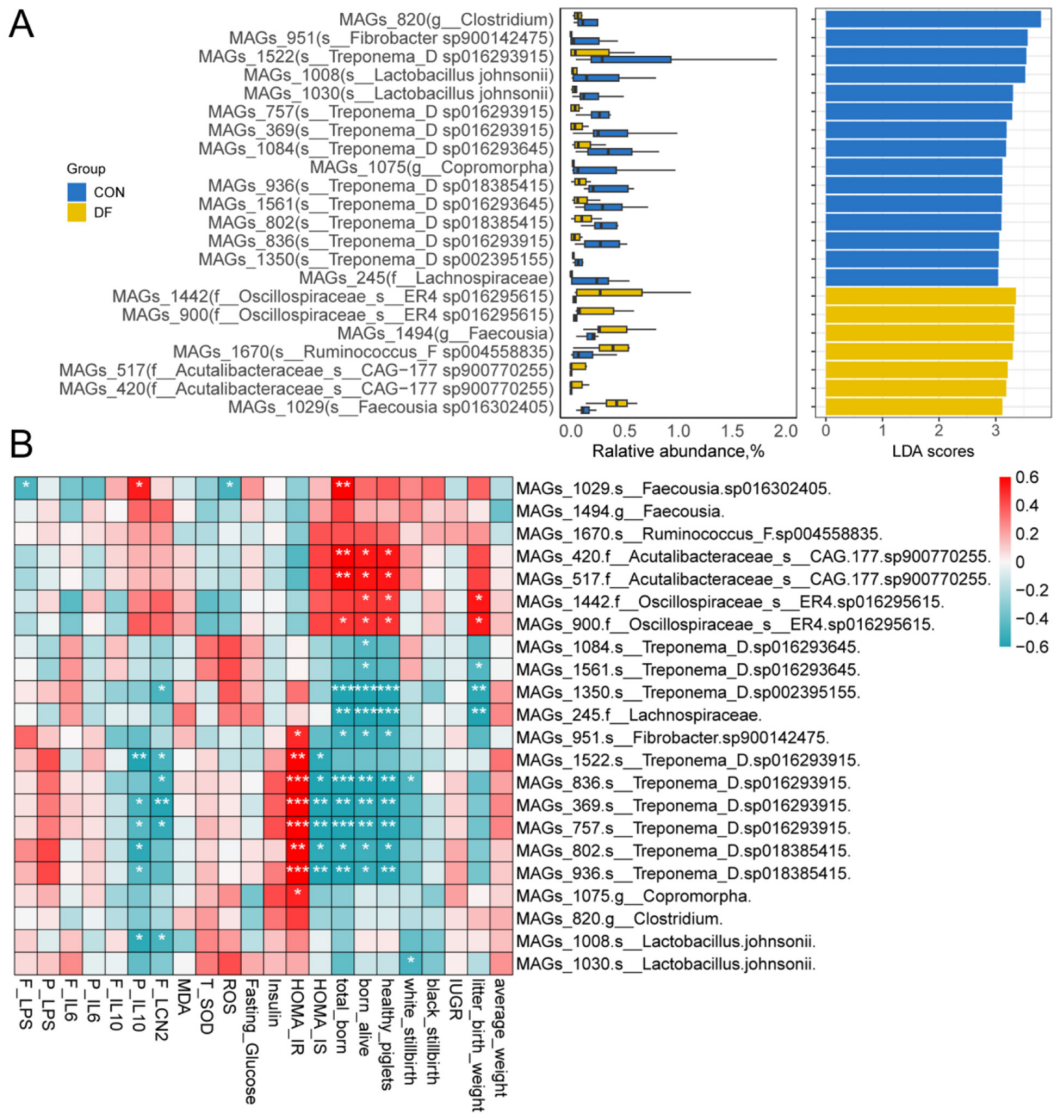
Identification of differential fecal microbial MAGs and their correlations with host physiological and reproductive phenotypes. **(A)** LEfSe analysis of differential MAGs (LDA scores > 3), **(B)** Relationships between the differential MAGs and fecal and plasma parameters, as well as reproductive performance of sows. CON, control diet; DF, functional fiber diet. Color intensity represents the correlation coefficient (ρ). Results are expressed as median and quartile. *P* < 0.05 indicates statistical significance (*P < 0.05, **P < 0.01, and ***P < 0.001).

**Figure 5:**
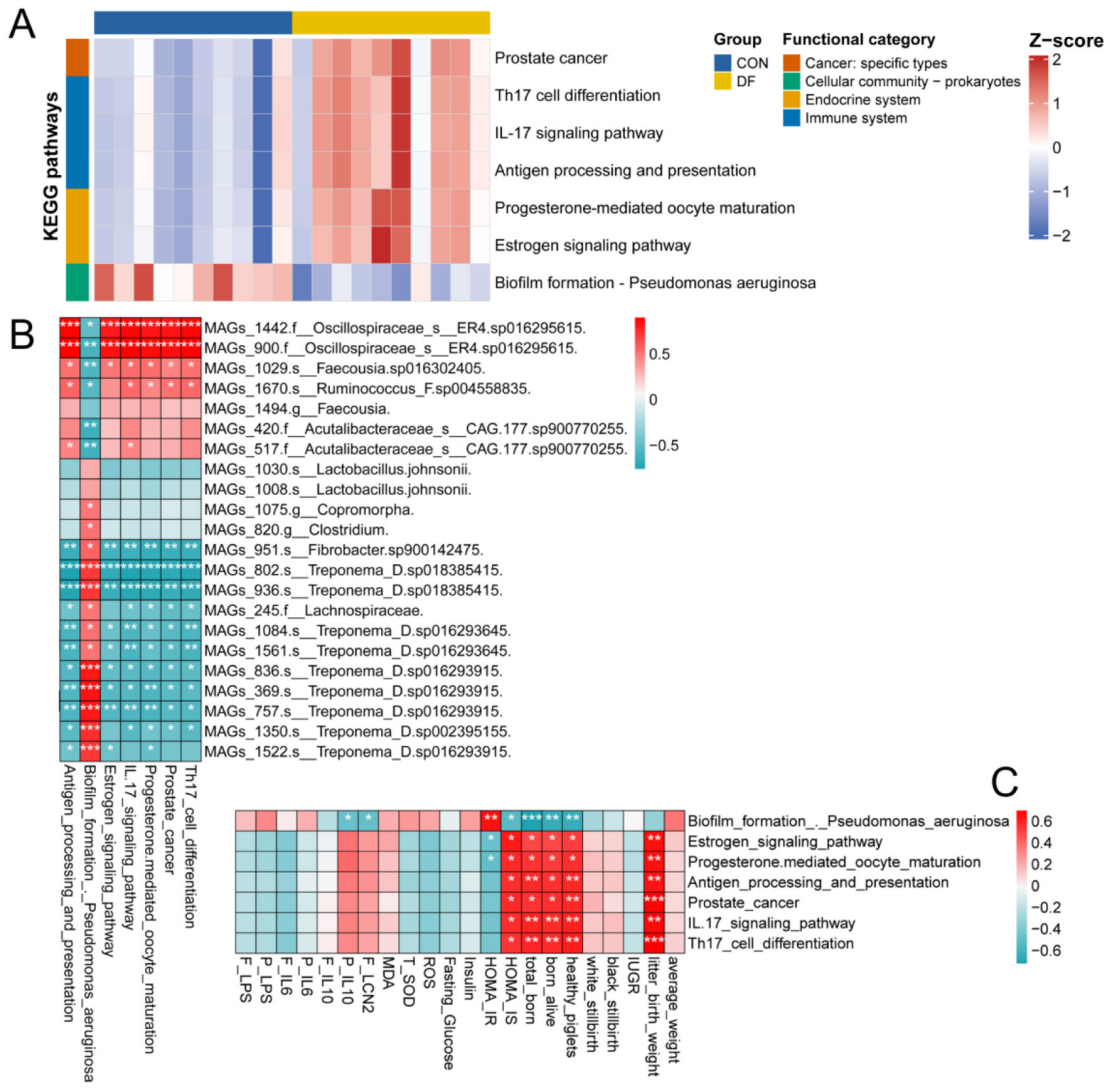
Alterations in fecal microbial functional pathways and their associations with specific MAGs and host phenotypes. **(A)** Heatmap illustrating the abundance (normalized by Z-score) of differential KEGG functional pathways between the CON and DF groups, **(B)** Relationships between the identified differential MAGs and the altered KEGG pathways, **(C)** Relationships between the altered KEGG pathways and host phenotypes. CON, control diet; DF, functional fiber diet. Color intensity represents the correlation coefficient (ρ). Results are expressed as median and quartile. *P* < 0.05 indicates statistical significance (*P < 0.05, **P < 0.01, and ***P < 0.001).

To understand the functional consequences of microbial community changes in sows, we conducted the metagenomics analysis and annotated microbial genes based on KEGG database. LEfSe analysis was performed on the 1,721 strain-level metagenome-assembled genomes (MAGs) to identify the microbes that were specific to each of the two groups (Supplementary Table S2). A total of 245 microbial MAGs were significantly different (Supplementary Table S2). As shown in Figure 4A, using an LDA score > 3 as the threshold, 15 MAGs were enriched in the CON group and 7 in the DF group. In the CON group, the MAGs belonging to genus *Treponema* were significantly enriched, whereas those affiliated with the family Oscillospiraceae were significantly enriched in the DF group (Figure 4A). These results are consistent with the differential analysis results of 16S rRNA gene sequencing. Generalized linear models were applied to analyze the critical microbial KEGG pathway (Figure 5A). Among the 428 pathways, 7 showed significant differences, including prostate cancer, Th17 cell differentiation, IL-17 signaling pathway, antigen processing and presentation, progesterone-mediated oocyte maturation, estrogen signaling pathway, and Biofilm formation—*Pseudomonas aeruginosa* (Figure 5A).

To further elucidate the potential interactions between microbial function and reproductive performance, spearman correlation analysis was conducted as shown in Figures 4B and 5B, C. Consistent with the 16S sequencing results, MAGs_1442 and MAGs_900 belonging to Oscillospiraceae showed a significant positive correlation with the numbers of total born, born alive and healthy piglets, as well as litter birth weight (Figure 4B). Similarly, MAGs_420 and MAGs_517 (Acutalibacteraceae) were positively correlated with the numbers of total born, born alive and healthy piglets (Figure 4B). In contrast, MAGs_836, MAGs_936, MAGs_369, MAGs_757, and MAGs_802 belonging to *Treponema* were all negatively correlated with reproductive performance. Regarding biochemical parameters, the MAGs of Treponema showed a significantly negative correlation with plasma IL-10 and fecal LCN2, while demonstrating a significantly positive correlation with HOMA-IR (Figure 4B).

Furthermore, we explored the relationships between specific MAGs and altered functional pathways (Figure 5B). Notably, the Oscillospiraceae (MAGs_1442 and 900) exhibited highly significant positive correlations (ρ > 0.8, *P* < 0.001) with the six aforementioned metabolic and immune-related pathways (e.g., antigen processing and presentation, Th17 cell differentiation), while being negatively correlated with biofilm formation. Conversely, the *Treponema* MAGs displayed the exact opposite trend, showing strong positive correlations exclusively with biofilm formation—*Pseudomonas aeruginosa* (Figure 5B).

Correlation analysis of key functional pathways with biochemical parameters and reproductive performance in sows revealed that the six pathways enriched in the DF group (including antigen processing and presentation, estrogen/IL-17 signaling pathways, etc.) were all significantly positively correlated with HOMA-IS, the number of total born, born alive, healthy piglets and litter birth weight (Figure 5C). In contrast, biofilm formation—*Pseudomonas aeruginosa* was significantly negatively correlated with plasma IL-10, fecal LCN2, HOMA-IS, the number of total born, born alive and healthy piglets (Figure 5C).

## 4 Discussion

This study aimed to evaluate how dietary supplementation with 1% functional fiber during gestation affects reproductive performance of sows and metabolic health, as well as gut microbiota. Our findings show that functional fiber improved the reproductive performance and metabolic health. This is accompanied by the changes in the composition and function of the microbiota.

### 4.1 The effects of functional fiber on reproductive performance in sows

In the present study, dietary supplementation with 1% functional fiber significantly increased the numbers of total born, born alive, healthy piglets and litter birth weight. Notably, although DF supplementation increased litter birth weight, no significant difference was observed in the average individual piglet birth weight, suggesting that the increased litter weight was primarily due to the greater number of piglets born rather than enhanced fetal growth per piglet. Gestation is a critical phase in the sow reproductive cycle (35), during which numerous factors—including metabolic homeostasis, progressive oxidative stress, and nutritional intake—can substantially influence reproductive performance (3, 36, 37). Wu et al. (38) reported the functional fiber can improve the birth weight of piglets and reduce the percentage of IUGR piglets. Increasing studies have demonstrated that dietary fiber supplementation in gestation diets enhances reproductive performance in sows (20, 39, 40). These benefits are closely linked to fiber's effects on improving maternal metabolic status, promoting the production of short-chain fatty acids (SCFAs), and modulating the gut microbiota.

### 4.2 The effects of functional fiber on inflammatory response in sows

Metabolic syndrome in sows is characterized by changes in maternal inflammatory and immune status during the perinatal period (3, 41). In the present study, dietary supplementation with DF significantly increased plasma IL-10 concentration, whereas plasma and fecal LPS, plasma and fecal IL-6, fecal IL-10, and fecal LCN2 were not significantly altered compared with the CON group. These findings suggest that DF may primarily enhance systemic anti-inflammatory capacity rather than broadly suppress pro-inflammatory signals or intestinal inflammatory markers. This selective modulation of anti-inflammatory responses is consistent with prior findings where dietary fiber supplementation increases IL-10 levels in serum, colostrum, or milk while variably reducing pro-inflammatory cytokines such as IL-6 and TNF-α (19, 42, 43). For example, sows fed sugar beet pulp significantly reduced serum IL-6 and TNF-α, while elevating serum IL-10 at day 110 of gestation (42). Liu et al. reported that alfalfa meal supplementation during gestation and lactation significantly reduced serum and fecal IL-6 and TNF-α while elevating IL-10 at day 100 of gestation (19). And Xu et al. reported that soluble fiber in pregnancy diet markedly reduced the levels of plasma IL-6 and TNF-α (43). The absence of a significant reduction in IL-6 in the present study may be due to differences in fiber type, the timing of sampling relative to peripartum inflammatory dynamics, or baseline inflammatory status. Notably, no significant differences were observed in fecal LPS, fecal IL-6, fecal IL-10, or fecal LCN2 between the two groups, indicating that the anti-inflammatory effect of DF was not clearly reflected in these fecal biomarkers under the present conditions.

### 4.3 The effects of functional fiber on oxidative status and insulin homeostasis in sows

Pregnancy in sows involves substantial physiological adaptations and exposure to environmental stressors, which commonly lead to progressive oxidative stress (41) accompanied by changes in insulin sensitivity (43). Recent studies have shown that gestation diet supplement with dietary fiber can alleviate oxidative stress and improves insulin sensitivity (43–47). In line with these findings, our study demonstrated that supplementation with 1% functional fiber during gestation significantly mitigated ROS and HOMA-IR, improved HOMA-IS in sows. Not all indicators responded uniformly, however. The lack of significant changes in MDA and T-SOD suggests that DF may primarily reduce reactive oxygen accumulation rather than broadly affect all oxidative stress-related markers under the present experimental conditions. Likewise, although fasting glucose and insulin were not significantly altered, the changes in HOMA-IR and HOMA-IS indicate an overall improvement in insulin responsiveness. Together, these findings, in agreement with Xu et al. (43), support a beneficial role of DF in maintaining oxidative and insulin homeostasis in gestating sows.

### 4.4 The effects of functional fiber on the fecal SCFAs in sows

Although dietary fiber is generally considered an important substrate for microbial fermentation and SCFA production, the present study showed that the functional fiber (DF) group had significantly lower fecal total SCFAs, particularly propionate and butyrate, than the CON group. This result may be partly explained by differences in fiber composition rather than total fiber level alone. According to the analyzed nutrient composition (Supplementary Table S1), the DF diet contained a markedly lower level of soluble fiber than the CON diet (0.53 vs. 1.44%), whereas insoluble fiber and total NDF were slightly higher in the DF diet. Since soluble fiber is generally more readily fermented by gut microbiota and serves as a major substrate for SCFA production (48), the reduced soluble fiber content in the DF diet may have limited microbial fermentation and thereby contributed to the lower SCFA concentrations observed in the DF group. Therefore, the present findings suggest that the fermentability and physicochemical characteristics of dietary fiber, especially the proportion of soluble fiber, may be more important than total fiber content *per se* in determining intestinal SCFA production.

However, fecal SCFA concentrations reflect the net balance among microbial production (49), intestinal absorption, and host or microbial utilization, rather than microbial production alone. Therefore, lower fecal SCFA concentrations do not necessarily indicate reduced overall fermentation efficiency or diminished physiological benefit to the host. In the present study, reproductive performance was improved in the DF group despite lower fecal SCFA concentrations, suggesting that the beneficial effects of functional fiber cannot be explained simply by increased fecal SCFA output. Instead, these benefits may be more closely related to DF-induced remodeling of the gut microbiome, including shifts in microbial composition, functional potential, and ecological stability during late gestation. This interpretation is consistent with previous findings showing that functional fiber can enhance microbial stability in late gestation (22). Together, these results indicate that the relationship among fiber composition, SCFA metabolism, and reproductive performance is complex, and that microbiota-mediated effects of DF may extend beyond SCFA production alone.

### 4.5 The effects of functional fiber on the fecal microbiota diversity in sows

Dietary supplementation with functional fiber substantially reshaped the fecal microbiota, as reflected by reduced richness-related α-diversity indices and clear separation in β-diversity between the two groups. Compared with the CON diet, the DF diet contained a lower level of soluble fiber, whereas insoluble fiber and total NDF were slightly higher. This difference in fiber composition may partly explain the reduced α-diversity observed in the DF group. Soluble fiber is generally more accessible to microbial fermentation and can provide a broader range of substrates for diverse microbial populations, whereas a lower proportion of soluble fiber may narrow the available ecological niche and promote the selective enrichment of microbial taxa that are better adapted to the prevailing substrate conditions (50, 51). Such selective enrichment may reduce community richness and evenness, leading to lower ACE, Chao1, and Observed species indices.

Reports on the effects of dietary fiber supplementation on gut microbiota α-diversity in sows during late gestation remain inconsistent. In the present study, α-diversity was significantly lower in the DF group. This is consistent with the findings of previous study (43). In contrast, several studies using fermentable soluble fibers reported increased alpha diversity (16, 18). Other investigations, however, observed no significant alterations in α-diversity metrics (17, 19). However, despite microbial composition and functional diversity increases typically reflecting enhanced community stability and resilience, which are generally advantageous for the host (52, 53). Other investigations have shown that dietary fiber interventions can selectively enrich certain species, thereby reducing overall species diversity and evenness (54). In the present study, the distinct separation in β-diversity further supports that functional fiber induced a substantial restructuring of the microbial community. Together with the functional metagenomic findings, these results suggest that functional fiber primarily drove a directed remodeling of the gut microbiota, rather than a generalized expansion of microbial diversity.

### 4.6 The effects of functional fiber on the fecal microbiota composition in sows

Firmicutes and Bacteroidota were the most dominant phyla, which is highly consistent with other studies (18, 43, 55). Notably, *Rikenellaceae_RC9_gut_group and Treponema* were identified as differentially abundant genera that were significantly enriched in the CON group and and were negatively associated with reproductive performance. *Rikenellaceae_RC9_gut_group* has been linked to fiber fermentation and the production of acetate, propionate, and succinate (56–58), and in the present study its abundance was positively correlated with fecal SCFA concentrations (Supplementary Table S3). However, despite the higher abundance of this taxon and higher fecal SCFA levels in the CON group, reproductive performance was inferior to that in the DF group. This finding suggests that increased SCFA output alone may not be the primary mechanism underlying the beneficial effects of DF. Instead, enrichment of *Rikenellaceae_RC9_gut_group* in the CON group may reflect a less favorable microbial configuration or metabolic imbalance, consistent with previous observations that its higher abundance was associated with poorer reproductive outcomes in gestating sows (59). Likewise, although Treponema includes fibrolytic members (60, 61), its excessive abundance has been associated with gut dysbiosis, inflammatory activation, and adverse reproductive outcomes, including stillbirth and reduced litter size (11, 62). In line with these reports, the negative association between Treponema abundance and reproductive performance observed here suggests that suppression of potentially unfavorable taxa may be an important component of the DF response.

In contrast, taxa enriched in the DF group, particularly members of the Oscillospiraceae family, may represent key microbial mediators linking DF to improved host metabolic and immune homeostasis. LEfSe analysis identified *UCG-005, Family_XIII_AD3011_group*, and *NK4A214_group* as biomarkers of the DF group, and these taxa showed positive associations with reproductive performance. Among them, *NK4A214_group*, an unclassified genus within Oscillospiraceae, is of particular interest because it has been associated with fertility, bile acid metabolism, feed efficiency, and fiber degradation (63–65). In pigs, this taxon has also been linked to higher fiber digestibility and was previously identified as an important contributor to gut microbial network stability during gestation (22, 65). Similarly, *UCG-005*, another Oscillospiraceae-related taxon, was positively associated with total litter size in the present study. Together, these findings suggest that DF may promote a more favorable microbial ecosystem characterized by enhanced fiber utilization, improved metabolic signaling, and greater community stability. Such microbial changes may in turn contribute to improved reproductive performance by influencing host energy metabolism, bile acid-related pathways, and immune balance rather than simply by increasing total SCFA production.

### 4.7 The effects of functional fiber on the microbial function in sows

To further determine whether the taxonomic shifts induced by DF were accompanied by functionally relevant microbial changes, we analyzed metagenome-assembled genomes (MAGs) and KEGG pathways in relation to host phenotypes. The MAG-based results were broadly consistent with the 16S data and, importantly, were closely linked to the metabolic and reproductive outcomes measured in this study.

Numerous MAGs belonged to *Treponema* were significantly enriched in the CON group and exhibited a negative correlation with sow reproductive performance. This pattern is consistent with the poorer litter outcomes in the CON group, aligns with previous findings of marked *Treponema* enrichment in sows with high stillbirth rates (11), and suggests that enrichment of these MAGs may represent a microbial feature associated with an unfavorable reproductive state. By contrast, MAGs_1442 and MAGs_900, both belonging to the family Oscillospiraceae, were enriched in the DF group and positively correlated with reproductive performance. Similarly, MAGs_420 and MAGs_517, classified within Acutalibacteraceae, also showed positive associations with reproductive traits. Acutalibacteraceae, a relatively novel family within the gut and rumen microbiota, participate in carbohydrate and sulfur metabolism and are considered to possess potential beneficial metabolic functions (66). Because the DF group simultaneously displayed improved insulin sensitivity, lower insulin resistance, reduced oxidative stress, and enhanced anti-inflammatory status, these positive taxon–phenotype relationships support the interpretation that DF promoted a functionally advantageous microbiome linked to improved maternal homeostasis and reproductive success.

At the functional level, immune-related pathways, including Th17 cell differentiation, IL-17 signaling, and antigen processing and presentation, were enriched in the DF group. Microbiota-driven immune pathways do not necessarily indicate pathological inflammation; rather, they may reflect active host-microbiota immune crosstalk and mucosal immune adaptation (67). Recent studies demonstrate that maternal gut microbiota remotely controls maternal-fetal immune tolerance to suppress placental inflammation and prevent fetal loss (68). This interpretation is supported by our phenotypic data showing an enhanced anti-inflammatory profile in DF-fed sows. Thus, in the present study, enrichment of these immune-related microbial functions was accompanied by improved immune status and better reproductive performance, suggesting that DF may support pregnancy outcomes partly through modulation of microbiota-associated immune homeostasis.

In addition, endocrine-related pathways such as estrogen signaling and progesterone-mediated oocyte maturation were also enriched in the DF group. Although these pathway annotations do not directly demonstrate endocrine regulation by the microbiota, their enrichment is notable because it coincided with improved litter performance in DF-fed sows. Together with previous evidence that gut microbes can influence steroid hormone metabolism and reproductive physiology (69–71), our findings support a potential link between DF-induced microbial functional remodeling and host reproductive regulation.

In contrast, the CON group showed enrichment of the biofilm formation—*Pseudomonas aeruginosa* pathway. Biofilm formation is a well-recognized virulence-associated strategy that enhances bacterial persistence, immune evasion, and resistance to host defenses (72, 73). In the gut ecosystem, biofilm-associated microbial communities may disrupt mucosal barrier integrity and promote chronic low-grade inflammation, thereby contributing to impaired metabolic homeostasis (74). Consistently, in our correlation analysis, this pathway was negatively associated with reproductive performance and IL-10 levels, but positively associated with insulin resistance and negatively associated with insulin sensitivity. These relationships directly connect the microbial functional signature of the CON group with the less favorable host phenotype observed in this study. Given the importance of insulin sensitivity and inflammatory control for maternal nutrient partitioning and fetal development during late gestation, enrichment of this biofilm-related pathway may represent a dysbiotic feature linked to impaired metabolic and immune homeostasis and, consequently, poorer reproductive outcomes.

Overall, our correlation analyses indicate that the beneficial effects of DF were associated not merely with shifts in microbial composition, but with coordinated changes in microbial taxa and functions that were directly linked to improved litter performance, enhanced anti-inflammatory status, lower oxidative stress, and improved insulin sensitivity. These findings strengthen the conclusion that functional remodeling of the gut microbiome is a key mechanism underlying the reproductive benefits of DF supplementation in sows.

## 5 Limitations

This study has several limitations. First, the associations among functional fiber supplementation, microbiota shifts, host metabolic and inflammatory responses, and reproductive outcomes are correlative, and therefore causal relationships cannot be confirmed. Second, fecal SCFA concentrations were used as indicators of microbial fermentation, but they may not fully capture intestinal SCFA production, absorption, or host utilization. Third, although metagenomic binning and KEGG analyses provided useful insights into microbial functional potential, these data do not directly reflect transcriptional activity or metabolite production, and taxonomic annotation of some key MAGs remained limited. In particular, several performance-associated MAGs could only be assigned to the family level, which restricted the identification of specific microbial species or strains involved. Future studies using causal validation and integrated multi-omics approaches are warranted to better define the mechanisms underlying the reproductive benefits of functional fiber in sows. Taken together, future research should place greater emphasis on defining causal microbiota-mediated mechanisms, identifying key functional fiber characteristics, and integrating host metabolic, endocrine, and immune responses to better explain how functional fiber improves reproductive efficiency in sows.

## 6 Conclusion

In conclusion, functional fiber supplementation improved reproductive performance in sows, as evidenced by increased numbers of total born, born alive, and healthy piglets. These benefits were accompanied by enhanced anti-inflammatory status, reduced oxidative stress, lower insulin resistance, and improved insulin sensitivity during late gestation. Multi-omics analyses further showed that functional fiber reshaped the gut microbiome toward a more favorable functional profile, characterized by enrichment of Oscillospiraceae-associated taxa and pathways related to immune regulation and reproductive hormone signaling, along with suppression of biofilm-related microbial functions. Together, these findings suggest that functional fiber enhances reproductive efficiency in sows, at least in part, through functional remodeling of the gut microbiome and the maintenance of metabolic and immune homeostasis.

## Data Availability

The raw sequencing data for both 16S rRNA gene sequencing and metagenomic sequencing generated in this study have been deposited in the NCBI Sequence Read Archive (SRA) under BioProject accession number PRJNA1364848 (https://www.ncbi.nlm.nih.gov/sra/?term=PRJNA1364848). The original contributions presented in this study are included in the article. Further inquiries can be directed to the corresponding author.
