# Supplementary material for: Functional fiber supplementation improves reproductive performance in sows by reshaping gut microbial functions related to immunity and metabolic health
Source: Front Vet Sci. 2026 May 7;13:1834008. doi: 10.3389/fvets.2026.1834008 (PMC13189749; doi:10.3389/fvets.2026.1834008)
Supplement: Supplementary file 4 [file Data_Sheet_1.docx]

**Supplementary information**


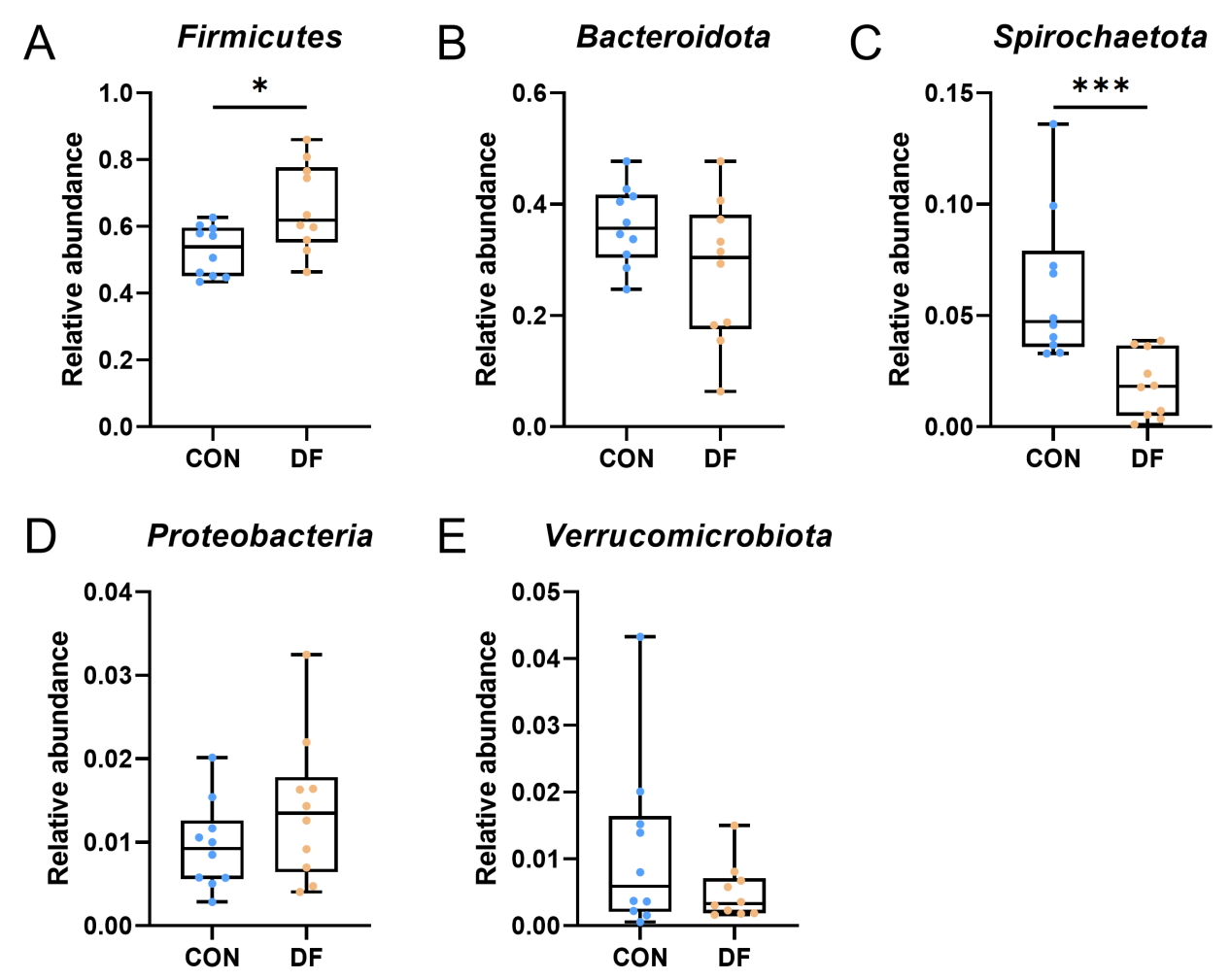


**Figure S1**. Differential analysis of top 5 phylum. The relative abundance of (**A**) *Firmicutes*, (**B**) *Bacteroidota*, (**C**) *Spirochaetota*, (**D**) *Proteobacteria*, (**E**) *Verrucomicrobiota*. Results are expressed as median and quartile. *P* < 0.05 indicates statistical significance (**P <* 0.05, ***P* < 0.01, ****P* < 0.001, and *****P* < 0.0001).


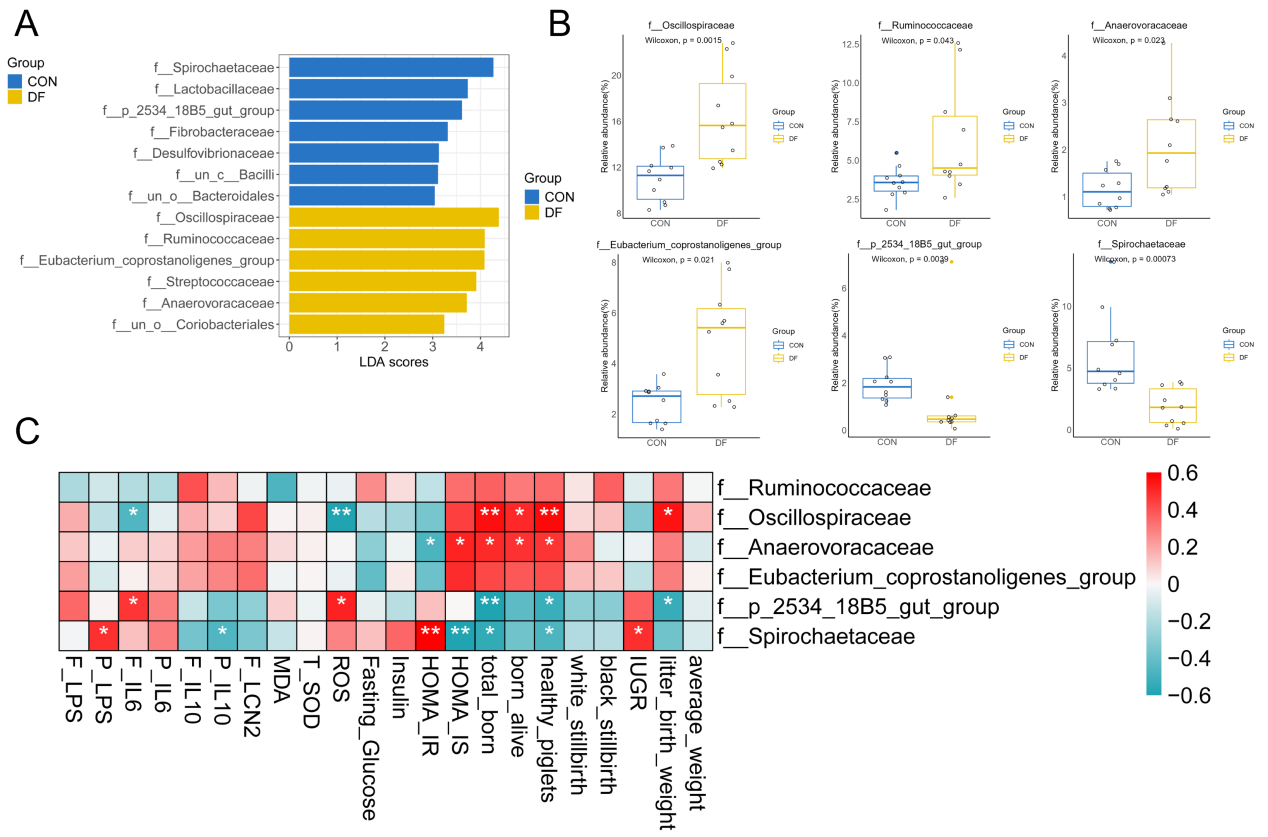


**Figure S2**. Differential analysis of fecal microbiota at the family level. (**A**) LDA distribution and LEfSe analysis identifying families with significant intergroup differences (LDA scores > 3), (**B**) Boxplot of 6 differential families with relative abundance > 1%, (**C**) Relationships between the differential taxa identified in panel B and fecal and plasma parameters, as well as reproductive performance of sows. Color intensity represents the correlation coefficient (ρ). Results are expressed as median and quartile. *P* < 0.05 indicates statistical significance (**P* < 0.05, ***P* < 0.01, ****P* < 0.001, and *****P* < 0.0001).
